# Strengthening Mechanism and Damping Properties of SiC_f_/Al-Mg Composites Prepared by Combining Colloidal Dispersion with a Squeeze Melt Infiltration Process

**DOI:** 10.3390/ma17071600

**Published:** 2024-03-31

**Authors:** Guanzhang Lin, Jianjun Sha, Yufei Zu, Jixiang Dai, Cheng Su, Zhaozhao Lv

**Affiliations:** 1 Key Laboratory of Advanced Technology for Aerospace Vehicles of Liaoning Province, Dalian University of Technology, Dalian 116024, China; yfzu@dlut.edu.cn (Y.Z.); jxdai@dlut.edu.cn (J.D.); sucheng@mail.dlut.edu.cn (C.S.); 2 State Key Laboratory of Structural Analysis, Optimization and CAE Software for Industrial Equipment, Dalian University of Technology, Dalian 116024, China; 3 School of Defence Science & Technology, Xi’an Technological University, Xi’an 710032, China; lvzhaozhao@xatu.edu.cn

**Keywords:** silicon carbide fiber, Al-based matrix composite, strengthening mechanism, mechanical properties, damping properties

## Abstract

SiC-fiber-reinforced Al-Mg matrix composites with different mass fractions of Mg were fabricated by combining colloidal dispersion with a squeeze melt infiltration process. The microstructure, mechanical and damping properties, and the corresponding mechanisms were investigated. Microstructure analyses found that SiC_f_/Al-Mg composites presented a homogeneous distribution of SiC fibers, and the relative density was higher than 97% when the mass fraction of Mg was less than 20%; the fiber–matrix interface bonded well, and no obvious reaction occurred at the interface. The SiC_f_/Al-10Mg composite exhibited the best flexural strength (372 MPa) and elastic modulus (161.7 GPa). The fracture strain of the composites decreased with an increase in the mass fraction of Mg. This could be attributed to the strengthened interfacial bonding due to the introduction of Mg. The damping capacity at RT increased dramatically with an increase in the strain when the strain amplitude was higher than 0.001%, which was better than the alloys with similar composition, demonstrating a positive effect of the SiC fiber on improving the damping capacity of composite; the damping capacity at a temperature beyond 200 °C indicated a monotonic increase tendency with the testing temperature. This could be attributed to the second phase, which formed more strong pinning points and increased the dislocation energy needed to break away from the strong pinning points.

## 1. Introduction

Aluminum-based matrix composites (AMCs) have a wide range of uses in the aerospace and transportation industries because of their high specific strength and stiffness, low coefficient of thermal expansion (CTE), and good resistance to corrosion [1,2]. Among different reinforcement approaches, carbon and silicon carbide (SiC) fibers have high priority to be used as reinforcements for AMCs due to their low density, large aspect ratio, excellent mechanical properties, low CTE, and high thermal and chemical stabilities [3,4]. Although carbon fiber is less expensive for AMC manufacturing and has a lower density than SiC fiber [5,6], the challenge is that the aluminum matrix cannot completely fill the carbon fiber bundles due to the poor wettability of the carbon fibers when combined with the aluminum [7]. Additionally, harmful interfacial reactions between carbon fibers and aluminum melt may take place during the fabrication process, which would damage the carbon fibers and lead to strong fiber–matrix interface bonding [8,9], resulting in low mechanical properties. As a result, SiC fibers are emerging as a better reinforcement solution for AMCs. Furthermore, the limited damping capacity of AMCs has restricted their use in some vibration-sensitive areas, such as in the structural components for space cameras, which require materials with favorable damping properties [10,11,12]. On the other hand, different mechanisms are responsible for the damping and strengthening of AMCs. Thus, it is essential to improve the mechanical properties and damping properties of AMCs simultaneously.

In recent years, great efforts have been made regarding the damping behavior of AMCs and various methods have been proposed to improve the mechanical properties and damping properties of AMCs [13,14,15,16,17]. Theo et al. [13] introduced different volume fractions of martensitic stainless steel and silicon carbide particles into Al-based matrix composites. They found that two-particle co-reinforced Al-Zn composites offered better damping and mechanical properties than those of SiC-particle-reinforced ones. Ram et al. [14] prepared an aluminum matrix composite with randomly distributed carbon fibers using the high-pressure infiltration method and investigated the effect of carbon fiber content on the damping capacity. The results showed that the peak damping of the composite firstly increased and then decreased with the increase in carbon fiber content, while the off-peak damping always increased with the increase in carbon fiber content. This phenomenon is believed to be related to the micro plasticity of the aluminum matrix and the dislocation breakaway at the interface.

As we know, the matrix is an important component for a composite, and its composition has a substantial impact on the properties of composites. Xu [18] selected the SiC fiber and used the vacuum pressure infiltration method to prepare continuous SiC-fiber-reinforced aluminum composites, where the volume fraction of the SiC fiber was designed to be 40% and different types of alloys (ZL102, ZL114A, ZL205A and ZL301) were used as the matrix. The effects of different matrix alloys on the interface structure, fiber damage, and fracture behavior were investigated. Chu et al. [19] investigated the influence of matrix categories on the damping capacity of SiC-fiber-reinforced aluminum matrix composites. The results revealed that the dominant damping mechanisms for SiC_f_/Al composites were dislocation damping at low temperatures (<150 °C) and grain boundary damping and interface damping at high temperatures (>150 °C). Hence, the microstructure of the matrix, such as the dislocation, grain size, the angle of the grain boundary, and the interface between the matrix and the fiber, essentially affected the damping capacity and dynamic modulus of composites across the whole temperature range. This work indicated that the damping capacity and mechanical strength in the SiC_f_/Al composites are not absolutely in conflict with each other, and can be achieved by adjusting the matrix composition. In terms of selecting the alloy composition of the matrix, it is evident that the introduction of Mg can not only improve the wettability of the SiC_f_ and Al matrix [20], but also strengthen the matrix. Li Z et al. found that the increase in Mg content was beneficial for the improvement of the damping properties of the alloy at room temperature, to a certain extent [21]. Therefore, the introduction of Mg in the matrix may have a positive effect on the improvement of both of mechanical properties and damping properties of SiC_f_/Al composites.

Therefore, it is of practical significance to study the effect of Mg content on the comprehensive properties of SiC_f_/Al-Mg composites. In this work, the SiC fiber and Al matrix doping with different Mg content were used to prepare the SiC_f_/Al-Mg composites. The SiC_f_/Al-Mg composites were prepared by combining the colloidal dispersion with the squeeze melt infiltration technique. The effects of Mg content on the microstructure, mechanical properties, and damping properties of SiC_f_/Al-Mg composites were studied.

## 2. Materials and Methods

### 2.1. Raw Materials

Al powders (average size: 50 μm, purity: >99.5%, supplied by Damao Co., Ltd., Tianjin, China) and Mg powders (average size: 100 μm, purity: >99.5%, supplied by Damao Co., Ltd., Tianjin, China) were used as the raw materials. SiC fibers (Cansas3203, supplied by Liyaxincai Co., Ltd., Quanzhou, China) with a length of about 500 μm and a diameter of 12 μm were used as the reinforcement to prepare SiC_f_/Al composites. To regulate the organization and properties of the SiC_f_/Al-Mg composites, Mg elements with mass fractions of 5%, 10%, 15%, and 20% were added.

### 2.2. Fabrication of SiC_f_/Al-Mg Composites

**Figure 1:**
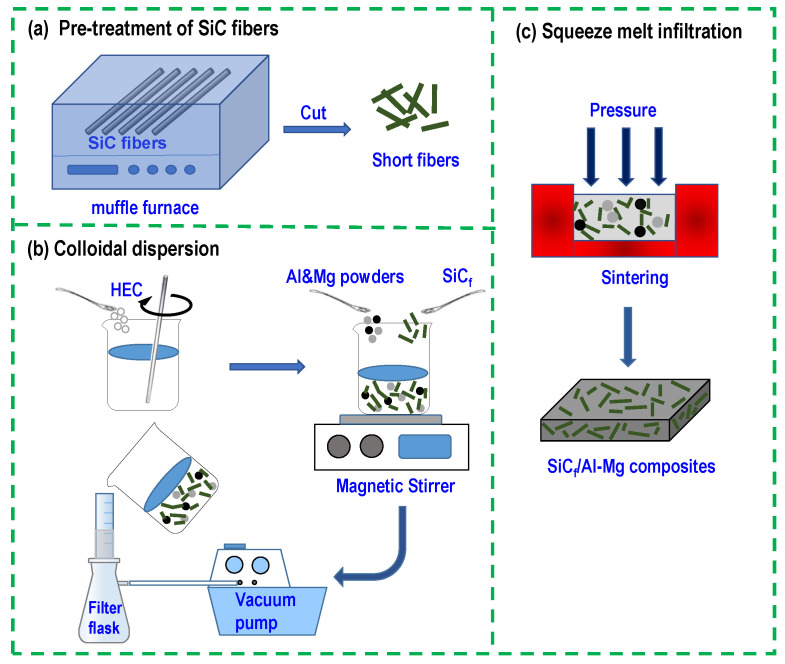
Schematic diagram of the preparation process of SiC_f_/Al-Mg composites.

Figure 1 shows the schematic diagram of the preparation process of SiC_f_/Al-Mg composites. Firstly, the SiC fibers were debonded by pre-treatment at 400 °C in a muffle furnace under atmospheric conditions, and then the debonded fibers were cut into short SiC fibers. Subsequently, a certain amount of hydroxyethyl cellulose (HEC) was dissolved in deionized water to form an HEC colloidal solution. Then, the short SiC fibers were poured into the colloidal solution and stirred for 20 min to fully disperse them in the HEC colloidal solution. The aluminum and magnesium powders were gradually poured into the colloidal solution dispersed with SiC_f_ and stirring was continued to make them fully dispersed.

The excess liquid was then extracted under negative pressure using a suction–filtration device to obtain a homogeneous mixture of SiC_f_, Al, and Mg powders. The mixture was moved into the mold and transferred into a vacuum-sintering furnace. When the furnace was heated to 680 °C, a pressure of 40 MPa was applied, and maintained for 15 min. Afterwards, the furnace was naturally cooled down to RT. Finally, a solidified block composite was obtained. The nominal volume fraction of SiC_f_ in the composite was 20%. The mass fractions of Mg in the Al matrix were 5%, 10%, 15%, and 20%, respectively. Correspondingly, they were labelled SiC_f_/Al-5Mg, SiC_f_/Al-10Mg, SiC_f_/Al-15Mg, and SiC_f_/Al-20Mg. In a control experiment, the same process was applied to prepare the SiC_f_-reinforced pure Al matrix composite labelled SiC_f_/Al.

### 2.3. Characterization

The density of the composites was measured using Archimedes’ method. The consisting phases were characterized by X-ray diffraction (XRD) (D/Max 2400, Rigaku Co., Tokyo, Japan). The Vickers hardness of the composites was tested on a Vickers hardness tester at RT with a load of 5 Kg for 10 s. The morphologies of the polished and fractured surfaces of the composites were analyzed using FE-SEM (NOVA NanoSEM 450, FEI, Hillsboro, OR, USA) equipped with an Energy Dispersive Spectrum (EDS). The strength was carried out on the test bars with 25 × 2.5 × 2 mm^3^ (length by width by thickness, respectively) using a 3-point flexural test at RT. At least three bars were used for each composite. Each test was loaded with a crosshead speed of 1 mm/min. The elastic modulus was obtained based on the strain measured by the strain gauge sensor.

The damping capacity of the composites were measured with single cantilever mode according to the standard test method: ASTM E756-05. The composites were machined into samples with dimensions of 1 × 5 × 30 mm^3^. The temperature dependence of the damping capacity was tested by dynamic mechanical analysis (DMA) (DMA-Q800, TA, New Castle, DE, USA) at temperatures ranging from RT to 400 °C with a heating rate of 5 °C/min, under a strain amplitude of 3 × 10^−4^ and a frequency of 1 Hz. The strain dependence of the damping capacity was also measured by DMA, with strains ranging from 1 × 10^−5^ to 2.5 × 10^−4^ at RT with a frequency of 1 Hz.

## 3. Results and Discussion

### 3.1. Microstructure

**Figure 2:**
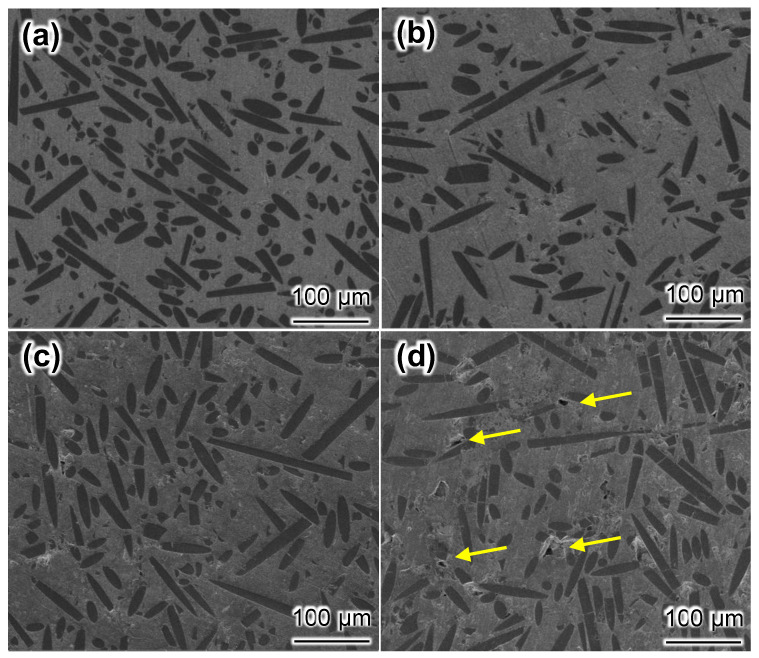
Morphologies of composites: (**a**) SiC_f_/Al-5Mg; (**b**) SiC_f_/Al-10Mg; (**c**) SiC_f_/Al-15Mg; (**d**) SiC_f_/Al-20Mg. Arrows in (**d**) denote pores located at surface.

Figure 2 shows the polished surface morphologies of composites. When the mass fraction of Mg was less than 20%, it was found that the composites had a dense structure and a homogeneous distribution of SiC fibers, and none of them showed evidence of SiC fiber aggregation (Figure 2a–c). This is because fibers in an aluminum matrix can be successfully dispersed via colloidal dispersion [22]. Figure 2d shows the morphology of the SiC_f_/Al-20Mg composite. As indicated by the arrows in the image, it was apparent that the composite contained pores. The pores in the SiC_f_/Al-20Mg composite could be attributed to the high Mg content. Furthermore, it was clear that the SiC fibers were bonded to the aluminum matrix and did not exhibit the presence of a reactive phase at the interface.

According to the aluminum–magnesium phase diagram, if the magnesium content in the aluminum matrix is high, the melting point of the alloy will be lowered, which results in the composites being unable to maintain sufficient pressure during solidification [20]. As a result, pores would occur in the composites. Additionally, the microscopic morphologies of the four composites revealed that SiC fibers exhibited good isotropy and no obvious orientation distribution, indicating that the current process produced composites with good isotropy.

In order to understand the relationship between the composites’ density and the Mg content clearly, the relative density of composites was calculated according to the ratio of the measured bulk density to the theoretical density. The results are shown in Table 1.

### 3.2. Phase Analysis

**Figure 3:**
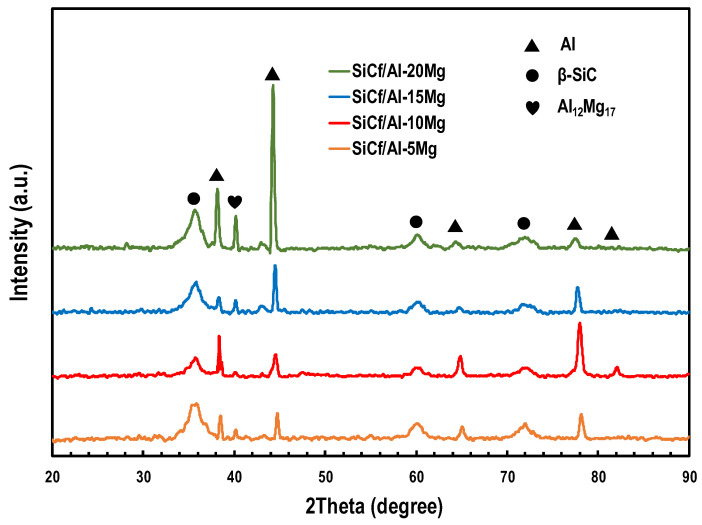
XRD patterns of the SiC_f_/Al-Mg composites.

Figure 3 shows the XRD spectra of SiC_f_/Al-Mg composites. It is clear from the spectra that each composite contains strong Al and SiC peaks, and an Al_12_Mg_17_ peak. The SiC peaks are situated at 2θ of 35.7°, 60.4°, and 71.8°, respectively. According to JCPDS cards (SiC: 49-1428), the three SiC peaks correspond to the (111), (220), and (311) crystallographic planes of β-SiC. The absence of the diffraction peaks of Mg in the XRD patterns might be attributed to the solid solution that formed with the Al matrix during the fabrication of the composites [23].

According to Bragg’s law, solid solution atoms in Al which are smaller than Al atoms will shift the position of the diffraction peaks of Al towards the lower diffraction angle. From Figure 3, it could be seen that the position of the Al peaks shifted to the low diffraction angle when the Mg element was introduced. Therefore, it can be inferred that Mg was dissolved into the Al matrix during the fabrication process. The existence of Al_12_Mg_17_ peaks demonstrated that Mg reacted with Al, and it can be seen that the Al_12_Mg_17_ peak in the pattern of SiC_f_/Al-20Mg was more prominent, which implies that more Al_12_Mg_17_ was generated in SiC_f_/Al-20Mg. It has been reported that SiC may react with the Al matrix at a high temperature to produce the reactants Al_4_C_3_ and Si [24]. However, in the current work, no XRD patterns showed the presence of the Al_4_C_3_ and Si phases. As we know, the Al_4_C_3_ phase is a brittle phase, and its presence is not conducive to the mechanical properties of the composite. This is probably due to the low preparation temperature, short processing time, and the existence of Mg. All these factors would restrict the reaction of SiC fiber with Al. Similar results were also found in the literature [20].

**Figure 4:**
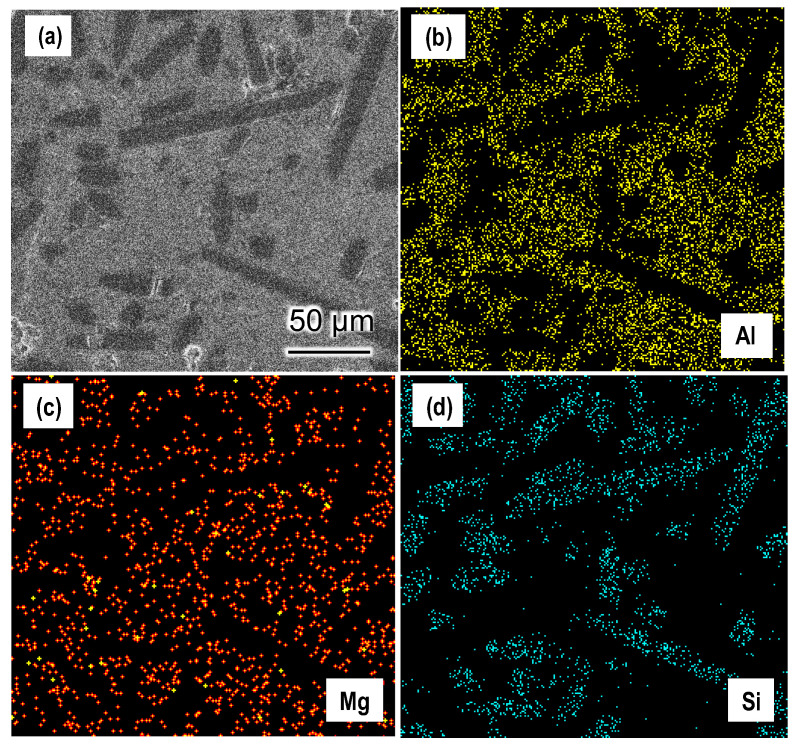
EDS analysis of the SiC_f_/Al-20Mg composite: (**a**) SEM image of the selected area; (**b**) mapping of Al element; (**c**) mapping of Mg element; (**d**) mapping of Si element.

In order to further analyze the distribution of different elements in the SiC_f_/Al-Mg composites, the selected areas of SiC_f_/Al-20Mg composites were examined by EDS surface scanning, as shown in Figure 4. Figure 4a shows the selected area and Figure 4b–d show the distribution of each element. It is clear from Figure 4b,c that the Mg co-existed with the Al element in the same area, implying that Mg was diffused into the Al matrix and formed a solid solution. This is consistent with the analysis of the XRD pattern (Figure 3). On the other hand, the distribution of the Si element (Figure 4d) matched with the profile of SiC fibers and no Si elements were present in the matrix. As a result, this may also indicate that the reaction between the SiC fiber and the Al matrix during the composites’ fabrication is not obvious.

### 3.3. Mechanical Properties

#### 3.3.1. Hardness

**Figure 5:**
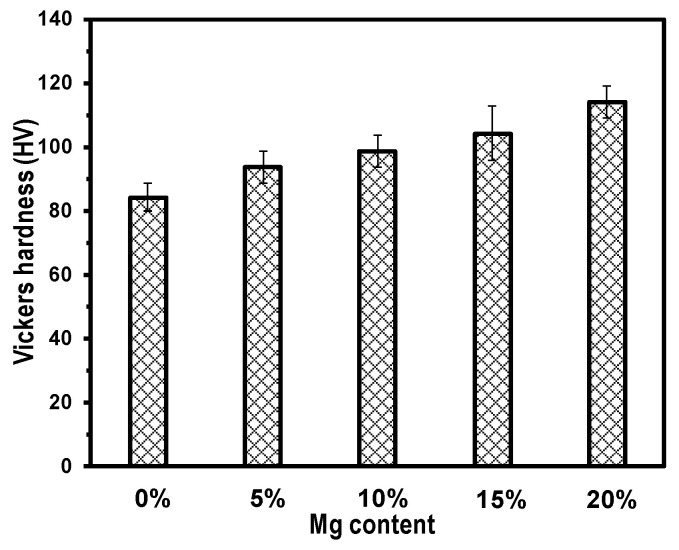
Vickers hardness of composites.

Figure 5 shows the Vickers hardness of SiC_f_/Al-Mg composites. The Vickers hardness of the composites increased with the increase in the mass fraction of Mg.

The Vickers hardness of the SiC_f_/Al-20Mg composite was 114.06 HV, which was 35.56% higher than that of SiC_f_/Al (84.14 HV). Obviously, the addition of Mg could improve the composite’s hardness due to the solution-strengthening effect [25]. Moreover, the precipitate phase Al_12_Mg_17_ could also play a role of dispersion strength.

#### 3.3.2. Flexural Strength

**Figure 6:**
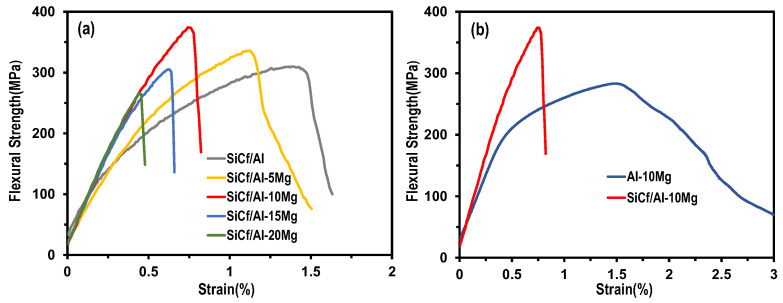
Mechanical properties of different composites: (**a**) stress–strain curves of SiC_f_/Al-Mg composites; (**b**) comparison of stress–strain curves between Al-10Mg alloy and the SiC_f_/Al-10Mg composite.

Figure 6 shows the stress–strain curves of different composites. The SiC_f_/Al composite exhibited the best plasticity, and the fracture strain was larger than 1.5% (Figure 6a). However, for the SiC_f_/Al-Mg composites, the fracture elongation decreased with the increase in Mg content. To clarify the effect of the SiC fiber on the mechanical properties of the composites, Figure 6b shows a comparison of the Al-10Mg alloy and the SiC_f_/Al-10Mg composite. The SiC_f_/Al-10Mg composites showed considerably higher flexural strength than those of the Al-10Mg alloy, while showing less apparent elongation. This might be attributed to the strong fiber–matrix interface bonding. SiC fibers are capable of carrying a large load, but they have a small deformation.

Table 2 summarizes the mechanical properties of SiC_f_/Al-Mg composites. It was found that the modulus of the SiC_f_/Al-Mg composites increased with the increase in Mg content, firstly. Moreover, it remained constant when the Mg content was beyond 10%. The modulus of the SiC_f_/Al-20Mg composite was 162.6 GPa, which is 41.6% higher than that of SiC_f_/Al. The interfacial bonding between the SiC fibers and the aluminum matrix can be strengthened by the addition of the Mg element, allowing for the full utilization of the SiC fibers’ load-bearing capability [19]. This meant that the composite’s modulus could be improved by the addition of Mg with a suitable content.

Compared to our previous work [22], the Young’s modulus of the SiC_f_/Al composites was much higher that of the Al matrix composites reinforced with SiC_p_ and C_f_ when the volume fraction of reinforcement was similar. Therefore, it can be considered that the introduction of Mg is conducive to improve the stiffness of Al matrix composites, with a better damping capacity expected. Again, it can be seen that the load-bearing capability of the SiC fiber was maximized when the Mg content exceeded 10%, and thus the modulus did not continue to increase with increasing Mg content. Correspondingly, the best flexural strength was achieved for the SiC_f_/Al-10Mg composites with a value of 372 MPa. The SiC fibers were evenly distributed throughout the matrix. The Mg element not only strengthened the interface bonding, but also the aluminum matrix, thus improving the flexural strength and the elastic modulus of the composite. However, when the Mg content was high, the pores started to form, which made the composites more easily breakable when subjected to external forces.

### 3.4. Fracture Morphology Analysis

**Figure 7:**
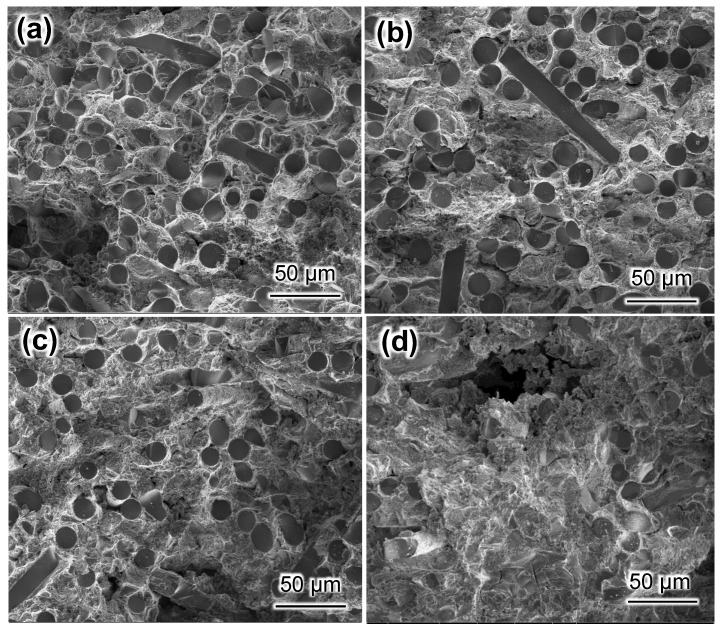
Fracture morphologies of the composites: (**a**) SiC_f_/Al-5Mg; (**b**) SiC_f_/Al-10Mg; (**c**) SiC_f_/Al-15Mg; (**d**) SiC_f_/Al-20Mg.

In order to better understand the effect of Mg content on the mechanical properties of SiC_f_/Al-Mg composites, the fracture morphologies of SiC_f_/Al-Mg composites were examined (Figure 7).

It could be seen that the fracture surfaces were uneven and the fibers were still embedded in the matrix, indicating that a strong interface formed between the fibers and the matrix. When the Mg content was low, some tearing edges, which are the symbol of ductile facture mode, were observed in SiC_f_/Al-5Mg, as shown in Figure 7a. When the Mg mass fraction was 10%, as shown in Figure 7b, it was found that the composite’s fracture surface presented few dimples, which suggests that the SiC fibers bonded strongly with the aluminum matrix. The strong interfacial bonding facilitated the transfer of loads. As a result, the composite exhibited excellent mechanical properties. As seen in Figure 7c,d, the composite fracture surfaces had porous flaws, which may reduce the mechanical capabilities of the composites. In particular, the pores were more obvious for the SiC_f_/Al-20Mg composite. Such pores would act as defects and cause stress concentration when the load was applied. If the stress intensity was very high, the cracks would first be initiated from the defects. Meanwhile, due to a strong interfacial bond between the fibers and matrix, the cracks’ propagation would not be inhibited and would penetrate directly into the fiber. As a result, low-stress damage occurred in the composites, as shown in Figure 6.

### 3.5. Damping Behavior

#### 3.5.1. Damping Capacity at Room Temperature

**Figure 8:**
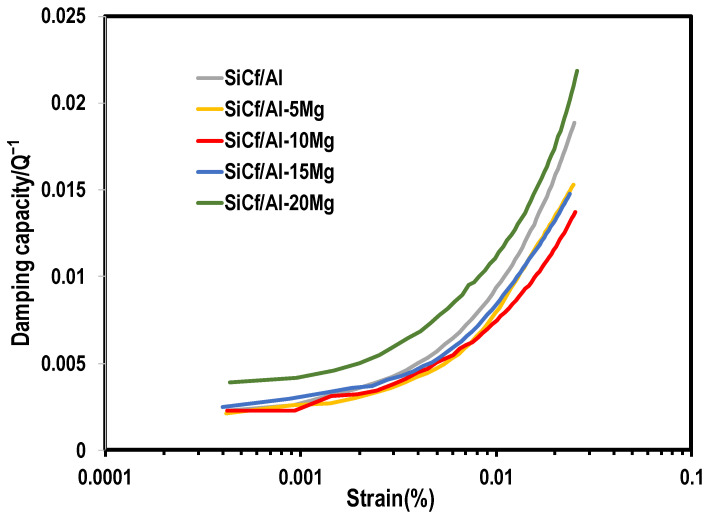
Strain dependence of the damping capacity for the SiC_f_/Al-Mg composites at room temperature.

The damping capacity as a function of strain is depicted in Figure 8. The damping capacity of all composites exhibited a weak dependence on the strain when it was lower than 0.001%. And then the damping capacity increased dramatically with an increase in the strain (higher than 0.001%). At a strain of 0.001%, the Q^−1^ values of SiC_f_/Al-5Mg, SiC_f_/Al-10Mg, and SiC_f_/Al-15Mg were around 0.003, respectively, and were about 0.004 for the SiC_f_/Al-20Mg. At a strain of 0.01%, all of the composites exhibited a Q^−1^ value of 0.007~0.011, which was higher than that for the alloys with similar composition [21], demonstrating that the SiC fiber had a positive effect on improving the damping capacity of the composite.

For the metal matrix composites at a low temperature regime, the dominant damping mechanisms could be associated with the consisting phases and the dislocation in the composite [26,27]. The contribution of the consisting phases to the damping can be described by the rule of mixing (ROM). The damping values of Al and SiC were 0.003 and 0.001 [19,28], respectively. The damping values of the SiC_f_/Al composite calculated by ROM were less than 0.003 in most of the test ranges of strain, which was lower than the experimental ones. This implies that the damping of the SiC_f_/Al composite should be related to other mechanisms. The large difference in CTE between the SiC fiber and the Al matrix would cause the accumulation of thermal stresses during the fabrication process, which would lead to the creation of dislocations in the matrix. It was considered that the dislocation also played a role in the damping capacity of SiC_f_/Al composites at RT. As for dislocation damping, the Granato–Lücke (G-L) model is widely accepted [29].

According to the G-L model, the dislocation in the material was similar to the elastic strings pinned in the weak points (such as solution atoms, vacancies, etc.) and the strong points (such as network nodes of dislocation, grain boundaries, the secondary phases, etc.). The dislocations sweep these pinning points and dissipate the vibration energy into the thermal energy irreversibly, which is the reason for the occurrence of dislocation damping. At a low strain amplitude, most of the dislocations were pinned by the weak pinning points and only oscillated in a small area, and thus the damping capacity was relatively low. When the strain reached a certain level, the snow-like breakaway of dislocations from the weak pinning point would happen. Thus, the area swept by dislocation segments would become larger. Consequently, when the strain was over a critical value, the damping capacity of the material increased dramatically with the increase in strain amplitude.

According to the G-L model, at a low strain amplitude, damping is dependent on the frequency and the strain. The strain-independent damping component symbolized as Q_0_ can be described by Equation (1), as follows [21]:

Q_0_~ρL_c_^4^ (1)

where ρ is the mobile dislocation density and L_c_ represents the average length of the dislocation segment between weak pinning points.

Mg atoms, as solute atoms, affected the damping capacity of the SiC_f_/Al-Mg composites in two main aspects. Firstly, Mg atoms acted as weak pinning points, which hindered the bowing movement of the dislocations. Secondly, an increase in Mg content led to a higher dislocation density in the matrix. According to Formula (1), when the strain amplitude is low, the strain-independent damping of the composites depends on the dislocation density in the matrix and the average distance between weak pinning points. As the Mg content in the matrix increases, a large number of dislocations are generated in the matrix, resulting in dislocation density ρ increasing. Meanwhile, the increase in solute atoms in the matrix will also shorten the average distance L_c_ of weak pinning points. The strain-independent damping capacity of the composites will be determined by the combined effect of these two factors.

From Figure 8, it can be seen that the damping capacity at a low strain amplitude for the composites with Mg content of 0%, 5%, 10%, and 15% was roughly similar. This proves that the damping capacity is not significantly affected by the increase in Mg content through the above-mentioned combined effect. However, it is worth noting that the strain-independent damping capacity of the SiC_f_/Al-20Mg composite was significantly improved compared to other composites. It was suggested by previous research [30] that the presence and homogeneous distribution of a large number of secondary phases were beneficial to improve the damping capacity of aluminum alloys at RT, due to the generation of a large number of movable dislocations around it. In the SiC_f_/Al-20Mg composite, abundant Al_12_Mg_17_ phases precipitated in the matrix, and were detected by XRD. It is reasonably believed that the Al_12_Mg_17_ phase may make a contribution to the improvement of strain-independent damping in the SiC_f_/Al-20Mg composite.

On the other hand, the strain dependent damping component Q_H_ conforms to formulae as follows [19]:

(2) $$Q_{H}^{-1}=\frac{C_{1}}{\varepsilon }\exp\left(-\frac{C_{2}}{\varepsilon }\right)$$

(3) $$C_{1}=\frac{{\rho F}_{B}L_{N}^{3}}{6{\mathrm{bEL}}_{C}^{2}}$$

(4) $$C_{2}=\frac{F_{B}}{{\mathrm{bEL}}_{C}}$$

where ε represents the strain amplitude; C_1_ and C_2_ are constants related to the properties of a material; ρ signifies the dislocation density; F_B_ is the binding force between dislocations and weak pinning points; E is the elastic modulus; L_C_ and L_N_ are the average dislocation distance between the weak pinning points and the strong pinning points, respectively; and b is the Burger’s vector. Through Equation (2), the formula can be transformed into the following form:

(5) $$\ln\left({\varepsilon Q}_{H}^{-1}\right)={\mathrm{lnC}}_{1}-\frac{C_{2}}{\varepsilon }$$

**Figure 9:**
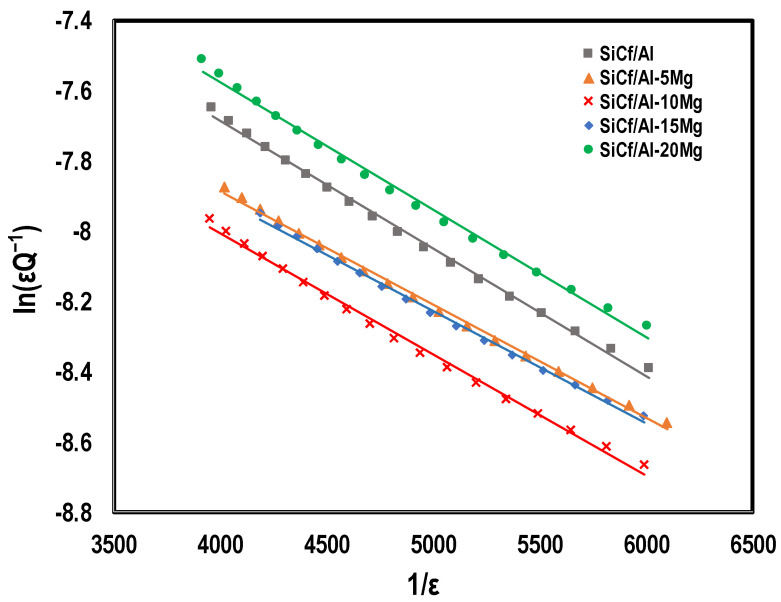
G-L plots of strain-dependent damping in SiC_f_/Al-Mg composites.

That is, if the strain-dependent damping of composites follows the G-L model, the plot of $\ln\left({\varepsilon Q}_{H}^{-1}\right)$ versus $\frac{1}{\varepsilon }$ should be satisfied with a linear relationship. In this work, the $\ln\left({\varepsilon Q}_{H}^{-1}\right)-\frac{1}{\varepsilon }$ plots obtained from the experiments are presented in Figure 9. It is clear that the favorable linear fit is exhibited. Hence, G-L theory is conformed for the composites in this work.

As the strain amplitude increased, the dislocations gradually began to break away from the weak pinning points and only engaged in a bowing motion within strong pinning points. From Formula (2), it can be seen that the strain-dependent damping increased with the average distance between the strong pinning points. For SiC_f_/Al-Mg composites, when the SiC_f_ content was constant, the distance between the strong pinning points was mainly dependent on the grain size of the matrix. However, an increase in Mg content would reduce the grain size of the matrix [31], thereby reducing the strain-dependent damping capacity of the composite. In the current work, for SiC_f_/Al-5Mg, SiC_f_/Al-10Mg, and SiC_f_/Al-15Mg composites, the dominant reason for the damping reduction could be attributed to the grain size refinement, as shown in Figure 8. However, the SiC_f_/Al-20Mg still exhibited a higher damping capacity than that of the SiC_f_/Al composite. This might be attributed to the effect of the second phase, mentioned earlier on, enhancing the damping mechanisms. In addition, a certain amount of flaws contained in SiC_f_/Al-20Mg composites may also contribute to the enhanced damping capacity.

#### 3.5.2. Damping Capacity at Elevated Temperatures

**Figure 10:**
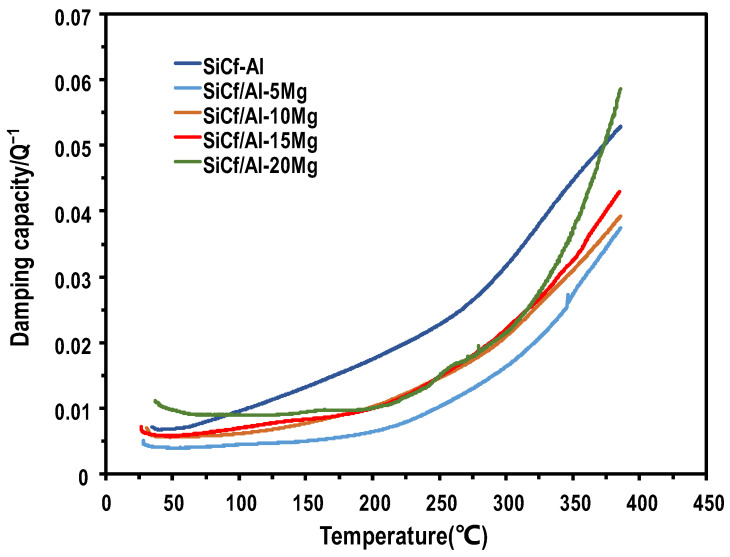
Temperature dependence of damping capacity in SiC_f_/Al-Mg composites.

Figure 10 shows the temperature-dependent damping capacity of the SiC_f_/Al-Mg composites. Obviously, the damping capacity for all composites exhibited a tendency for monotonic increase with the testing temperature. Such a phenomenon was also observed in other works [32,33,34]. Furthermore, the damping capacity of SiC_f_/Al-Mg composites is lower than that of SiC_f_/Al and shows no significant increase until 200 °C. This may be attributed to the second phase, which formed more strong pinning points and increased the dislocation energy needed to break away from new pinning points.

Furthermore, there was no evident damping peak in the curve of composites with low Mg content. Notably, SiC_f_/Al-15Mg exhibited one damping peak at 130 °C and SiC_f_/Al-20Mg exhibited two damping peaks at 165 °C and 262 °C, respectively. According to a previous work [31], the damping peak in metal matrix composites appearing at low-temperature regimes was associated with dislocation damping, while it was explained by grain boundary damping and interface damping mechanisms at high-temperature regimes (>200 °C). It was found that Mg introduction could decrease the stacking fault energy markedly and create dislocations and stacking faults in the Al matrix [21]. Thus, the occurrence of the damping peaks in SiC_f_/Al-15Mg and SiC_f_/Al-20Mg composites could be associated with the increase in the dislocation density caused by the high Mg content. The dislocation was the source of the damping capacity.

Moreover, the introduction of Mg refined the grain size of the matrix and led to an increase in the grain boundary area. When the temperature rose up to a high level, a softening of the matrix happened and viscous sliding between grain boundaries occurred. The grain boundary sliding dissipated more friction energy into thermal energy and led to a pronounced increase in the damping capacity, as well as the formation of a damping peak. It was also found in Figure 10 that the damping capacity increased with increasing Mg content when the temperatures were below 200 °C and above 300 °C. Based on the above analyses, the increase in the damping capacity of composites with Mg content with a low-temperature regime might be associated with the increased dislocations and stacking faults; the increase in the damping capacity of composites with Mg content at high-temperature regimes might be driven by the increased grain boundary area.

## 4. Conclusions

SiC-fiber-reinforced Al-Mg matrix composites with various levels of Mg content are fabricated via combining colloidal dispersion with a squeeze melt infiltration process. The effect of Mg content on the mechanical properties and damping capacity are investigated. The results are summarized as follows:

(1) SiC_f_/Al-Mg composites exhibit a homogeneous distribution of SiC fibers and a high relative density (higher than 97%) when the mass fraction of Mg is less than 20%. Mg could dissolve into the Al matrix, forming the Al_12_Mg_17_ precipitate phase. Fibers are well bonded with the Al-Mg matrix and no obvious reactive phase is present at the fiber–matrix interface.
(2) The Vickers hardness of the composites increases with increasing Mg content, and the highest value is 114.06 HV for SiC_f_/Al-20Mg, which is 35.56% higher than that of SiC_f_/Al. The enhanced hardness relates to the strengthening effect caused by the introduction of Mg.
(3) SiC_f_/Al-10Mg has the best flexural strength and elastic modulus, 372 MPa and 161.7 GPa, but the fracture elongation of the composites decreases with the increase in Mg content. This could be attributed to the strengthened interfacial bonding by the introduction of the Mg element.
(4) The damping capacity of SiC_f_/Al-Mg shows a weak dependence on the strain when the strain amplitude is lower than 0.001%. And then the damping capacity increases dramatically with an increase in the strain (higher than 0.001%), which is better than the alloys with similar composition, demonstrating that the SiC fiber has a positive effect on improving the damping capacity of the composite.
(5) The temperature-dependent damping capacity of the SiC_f_/Al-Mg composites reveals that all composites exhibited a tendency towards monotonic increase with testing temperature. Such an increase is more obvious at temperatures beyond 200 °C. This is attributed to the second phase, which forms more strong pinning points and increases the dislocation energy needed to break away from new pinning points.

## Figures and Tables

**Table 1:** Density of the SiC_f_/Al-Mg composite (g/cm^3^).

Composite	SiC_f_/Al	SiC_f_/Al-5Mg	SiC_f_/Al-10Mg	SiC_f_/Al-15Mg	SiC_f_/Al-20Mg
Bulk density	2.59	2.62	2.59	2.53	2.41
Relative density	96.64%	98.36%	98.27%	97.27%	94.21%

**Table 2:** Mechanical properties of the SiC_f_/Al-Mg composites.

Composite	Young’s Modulus (GPa)	Flexural Strength (MPa)
SiC_f_/Al	114.8 ± 3.6	309 ± 9
SiC_f_/Al-5Mg	125.1 ± 1.0	324 ± 12
SiC_f_/Al-10Mg	161.7 ± 2.0	372 ± 16
SiC_f_/Al-15Mg	160.3 ± 1.7	331 ± 10
SiC_f_/Al-20Mg	162.6 ± 5.6	283 ± 19

## Data Availability

The data presented in this study are available on request from the corresponding author due to privacy.
